# Exploratory study of the reproducibility of the SCore for INtrinsic and EXtrinsic skin aging (SCINEXA) scale in “Ruta Escondida de la Mitad del Mundo”, Ecuador, 2017

**DOI:** 10.1186/s12895-018-0078-9

**Published:** 2018-11-06

**Authors:** Martha Fors, Santiago Palacios, Kirsten Falcon, Karla Ventimilla, Lilia Simbaña, Carlos Lagos, Nélida Lasso, Carlos Navas

**Affiliations:** 1 Redondel del Ciclista, Universidad de Las Américas, Antigua Vía a Nayón, Quito, Ecuador; 2 Quito, Ecuador; 3 Ministry of Public Health, Quito, Ecuador

## Abstract

**Background:**

Few studies have been published related to the analysis of different skin aging parameters for whole-body skin using the SCINEXA scale for skin damage. The aim of this study was to evaluate the reproducibility of the SCINEXA scale (SCore for INtrinsic and EXtrinsic skin Aging) in South-Americans non-Caucasian population of a region of Ecuador.

**Methods:**

Exploratory observational study. Thirty subjects of both genders, over 40 years old and living in a rural area with particular characteristics regarding sun exposure were included. The SCINEXA scale was applied at three different time points to assess its reproducibility. Repeated measures analysis of variance was used for comparison of mean SCINEXA scores. Intraclass correlation coefficient, 95% CI and “Cronbach’s alpha” coefficient were performed to measure reproducibility.

**Results:**

Among participants, 86.7% were female; mean age was over 67 years old, with mainly low educational level, and almost half had more than six hours of sun exposure per day. Test-retest reproducibility of this scale demonstrated almost perfect agreement. The SCINEXA score was greater than 2 points in half of the subjects, reflecting aging due to sun exposure.

**Limitations:**

Most participants were women from one town in a particular geographical area, and the sample size was small. Genetic determinants of skin phenotypes were not assessed.

**Conclusions:**

The SCINEXA score is reproducible in South American non-Caucasian subjects of a particular region of the country. Damage from sun exposure was evident in participants.

## Key findings

- SCINEXA is a reproducible scale for subjects of a particular region of Ecuador who are non-Caucasians.
- Almost perfect agreement between observers.
- Most of the subjects in this study have skin damage due to sun exposure

## Background

Chronic exposure to solar radiation is the main environmental cause of skin aging and is responsible for the formation of wrinkles, loss of the ability of the skin to stay hydrated, appearance of pigmentary changes and decreased elasticity and tone; hence, areas exposed to the sun, such as the skin of the face and hands have much more severe photoaging characteristics than non-exposed areas.

The appearance of malignant and premalignant skin lesions is also sun- exposure related, and these lesions constituted the second highest prevalent group of neoplasms, for both males and females, in the population of the city of Quito, Ecuador [1].

The data are limited because there are not enough studies in Ecuador regarding photo damage, despite the city being in one of the regions with the highest exposure to ultraviolet radiation. It is also important to recognize that the vast majority of studies from other countries were carried out in Caucasians or Asians.

Skin aging is a complex biological process that can be classified by intrinsic and extrinsic aging [2]. Some descriptive skin-aging scales have been developed to assess skin aging signs, but Vierkotter et al. [3], published the results of the development of the SCINEXA scale (SCore for INtrinsic and EXtrinsic skin Aging), which measures these intrinsic and extrinsic factors regarding chronological skin aging and aging due to chronic exposure to ultraviolet (UV) radiation, respectively.

Validated tools are essential to evaluate skin aging for both dermatological and cosmetic research. SCINEXA is an already validated scale that is composed of 18 skin symptoms that are highly characteristic of extrinsic skin aging and five skin symptoms that are indicative of intrinsic skin aging. This scale has been validated in 74 subjects and represents an instrument that is easy to use, is non-invasive and is a sensitive tool for the simultaneous assessment of intrinsic and extrinsic skin aging. The SCINEXA scores range from 0 to 69, intrinsic SCINEXA from 0 to 15 and extrinsic SCINEXA from 0 to 54 [3].

Test–retest reproducibility is a method of estimating a tool’s reproducibility by administering it to the same person or group of people, in the same way, on two or more different occasions, hours or days apart [4].

Ecuador is located on the equatorial line, latitude and longitude 2°South and 77° 30° West, respectively. Quito, its capital, is located at the coordinates 0° 15° South and 78° 35° West and its altitude is recorded as from 1533 m to 3777 m above sea level. The urban area has an average altitude of 2850 m, and satellite data indicate that the Index of Ultraviolet Radiation (IUVR) in Quito during half of the day is on average 16, and there are records above 24. The city is located in the geographical area with the highest environmental risk, as described by all the scientific evidence, since the dose of ultraviolet radiation exceeds what is tolerable for humans, according to several sources. The so-called “hidden route of half of the world” is a well-defined geographic population whose rural habits make it the ideal place to investigate sun exposure-related skin damage.

In Ecuador, this scale has not yet been widely used. In the present study, we applied the SCINEXA scale at three different points of time to measure the ability of this scale to detect the magnitude of change over time in Ecuadorian subjects.

### Study design

This study is observational (cross-sectional exploratory study) and was performed over a period of three months (May–July 2017).

### Participants

Thirty volunteer subjects were recruited (40 to 80 years old, living in a rural area of Quito, both sexes). We performed a baseline evaluation and a retest at four days and at six weeks after the first assessment, which reached 90 evaluations. Each patient was evaluated by at least two different investigators who were blinded to the scores of the other investigators. After baseline assessment, a clinical standardization and formal training were set; this sample is part of a larger ongoing study.

We define ex-smoker an adult who has smoked at least 100 cigarettes in his or her lifetime but who had quit smoking at the time of interview. Unemployment was defined as people who do not have a job, have actively looked for work in the past four weeks, and are currently available for work. “Other” as category comprised those retired people or having informal jobs.

Habits of exposure to sun and prevention was been evaluated in daily basis.

Five cutaneous signs were evaluated: uneven pigmentation, fine wrinkles, lax appearance, reduced fat tissue, and seborrheic keratosis, which are all indicative of intrinsic skin aging or chrono-aging (intrinsic SCINEXA score).

We evaluated 18 signs of extrinsic aging of SCINEXA score: sunburn, freckles, actinic lentigo, hyperpigmentation and/or melasma, change in phototype, yellowness, pseudoscars, coarse wrinkles, solar elastosis, cutis rhomboidalis, elastosis, comedones and cysts of Favre-Racouchot, xerosis, teleangectasias, permanent erythema, actinic keratosis, basal cell carcinoma, squamous cell carcinoma and malignant melanoma (extrinsic SCINEXA score). Each item was scored as 0 (none), 1 (mild), 2 (moderate) or 3 (severe). For some items (uneven pigmentation, cutis rhomboidalis nuchae, elastosis, comedones and cysts of Favre Racouchot, and malignant skin tumors, a binary scale was used: (yes) or (no). Maximun possible score is 54.

### Sample size

The sample size calculation was derived from Walter et al. [5]. It was used a fixed alpha of 0.05, statistical power of 0.80 from three observations per subject with reliability values of R_0_ (Intraclass correlation coefficient) = 0.6 (moderate/acceptable) and R_1_ = 0.8 /substantial (expected), indicating a minimum sample size of *n* = 30. (drop-outs of 5%).

### Statistical analysis

Baseline characteristics of subjects were described through descriptive statistics. Absolute and relative frequencies were calculated for categorical variables, and the mean and standard deviation were calculated for quantitative variables. For comparison of proportions, Fisher’s Exact test was used because sample size was small and expected frequencies of cells were less than five. All tests were 2-sided, and significance was considered p less than 0.05.

Comparison of means at different time points was done through analysis of variance (repeated measures ANOVA).

For internal consistency, we calculated “Cronbach’s alpha” coefficient, which was interpreted as good if the estimate was more than 0.7.

Intraclass correlation coefficient (ICC) estimates and their 95% confident intervals were calculated based on mean-rating (*k* = 3), consistency, and a repeated measures 2-way mixed-effects model. ICCs were interpreted in conformity to the reference as slight (0.000 to 0.200), fair (0.201 to 0.400), moderate (0.401 to 0.600), substantial (0.601 to 0.800), or almost perfect (0.801 to 1.000).The significance level was set at *p* < 0.05.

For each subject, we calculated the mean SCINEXA score value, which was calculated as the ratio of extrinsic score to intrinsic score. These items were used to define an index that allowed differentiating between intrinsic versus extrinsic skin ageing. Two or more points in the SCINEXA score represents skin damage due to the sun and a score less than two indicates damage due to age. Lower SCINEXA scores correlate with less signs of aging, whereas higher scores mean greater signs of aging.

SPSS statistical package (SPSS (IBM Corp. Released 2013. IBM SPSS Statistics for Windows, Version 24.0. Armonk, NY, USA: IBM Corp.)) was used for data processing.

The Declaration of Helsinki Principles were followed, and all study subjects were informed in detailed written form and gave written consent. The ethics Committee of Universidad de Las Americas, Quito, Ecuador (CEISH-UDLA) approved the research protocol. We followed the SROBE guidelines for the report of this work. Universidad de Las Americas funded this research. There were no missing data.

## Results

The study included 30 subjects of both genders. Participant ages ranged from 44 to 84 years, with a mean ± SD of 67.7 ± 10.5 years. Most participants were women, and 83.3% of the respondents had completed only primary education.

90% of subjects were non-smokers and 96.7% were non-alcohol drinkers. Occupationally, 76.7% of participants were housekeepers and 16.7% were working in agriculture.

Most participants were of mixed race (86.7%). We found significant differences. Baseline characteristics of all participants are shown in Table 1.

**Table 1:** Baseline characteristics of included subjects

Age		
67.7(10.5)	Mean (SD)	
44–84	Min-MAX	
Skin phototype		*P* values*
Phototype II	1(13.3%)	*p* < 0.05
Phototype III	15(46.7%)	
Phototype IV	14 (50.0%)	
Gender		
Men	4 (13.3%)	*p* < 0.05
Women	26 (86.7%)	
Education		
None	0 (0.0%)	*p* < 0.05
Primary	26 (86.7%)	
Secondary	4 (13.3%)	
Smoking		
Non smoker	27 (90.0%)	*p* < 0.05
Ex-smoker	2 (6.7%)	
Smoker	1 (3.3%)	
Occupation		
Housekeeper	23 (76.7%)	*p* < 0.05
Agricultural worker	5 (16.7%)	
Unemployed	1 (3.3%)	
Other	1(3.3%)	

*Exact Fisher test. Source: Prepared by the authors from the study results

Half of the participants were exposed to the sun for more than 6 h per day. Most of these did not use sun protection. Of those who used protection, only 4 subjects always use sun protection. (Table 2). There were significant between-group differences.

**Table 2:** Habits of exposure to sun and prevention

Sun exposure		*P* values**
Less than 3 h a day	8 (26.7%)	*p* < 0.05
From 3 to 6 h a day	7 (23.3%)	
More than 6 h a day	15 (50.0%)	
Use of sun protector^a^		
Yes	14(46.7%)	*p* < 0.05
No	16 (53.3%)	
Frequency of sun protector^a^		
Never	16(53.3%)	*p* < 0.05
Hardly ever	6 (20.0%)	
Often	4 (13.3%)	
Always	4 (13.3%)	

^a^Daily basis

**Fisher exact test Source: Prepared by the authors from the study results

No patients were lost to follow-up; all patients completed the evaluations at four days and at six weeks (retest). We did not find significant statistical differences among the intrinsic, extrinsic domains and global means of SCINEXA scores at different time points (*p* > 0.05). (Table 3).

**Table 3:** Comparison of mean values of SCINEXA Score

	Baseline mean (SD)	Retest 4 days mean(SD)	Retest 6 weeks mean (SD)	*P* value
Mean of Intrinsic domain	6.80 (1.90)	6.80 (1.95)	6.90(2.20)	0.18*
Mean of Extrinsic domain	16.23 (3.99)	16.26 (4.38)	16.36 (4.90)	0.67*
Total score	23.03 (5.97)	23.06 (6.28)	23.26 (6.46)	0.33*

ANOVA (repeated measures)

*non-significant *p* > 0.05 Source: Prepared by the authors from the study results

According to SCINEXA index that classifies subjects in those with intrinsic aging (due to age) and those with extrinsic aging (due to sun), it was observed that the percentage of patients with more than two points in this index was approximately 50%. Half of the patients at 4 days and at the six-week assessment were also classified with skin damage because of sun exposure (Table 4).

**Table 4:** Number of subjects according SCINEXA Index

	Subjects with less than 2 points No. (%)	Subjects with more than 2 points No. (%)
Baseline	15 (50.0)	15(50.0)
Retest 4 days	15 (50.0)	15 (50.0)
Retest 6 weeks	13 (43.3)	17(56.7)

Source: Prepared by the authors from the study results

Cronbach’s alpha = 0.99 and 0.90 for intrinsic and extrinsic domains, respectively, of the scale (*p* < 0.05), indicating a very good internal consistency. Test-retest reproducibility, using the intraclass correlation coefficient, was substantial for both SCINEXA scale domains and in general (Table 5).

**Table 5:** Intraclass correlation coefficient and Cronbach’s alpha coefficient according to SCINEXA score domain

	ICC	IC95%	*P* value	Cronbach alpha
Intrinsic domain	0.98	0.97–0.99	0.000*	0.99
Extrinsic domain	0,75	0.60–0.86	0.000*	0.90
Total score	0.98	0.97–0.98	0.000*	0.99

*significant *p* < 0.05 Source: Prepared by the authors from the study results

## Discussion

Events linked to chronological aging (intrinsic) and extrinsic aging due to sunlight exposure are of major importance currently. The present study evaluated 30 volunteers living in rural areas whose lifestyles demand exposure to sun due to their agriculture activities.

Most of the participants of this study were housekeepers although they still work outdoors exposed to sunlight. It has been widely demonstrated that exposure to sun is an environmental factor that produces skin aging [6]. Participants in this study were not similar to those described in other studies. In a study performed by Perner et al. [7] was shown that a significant part of the ethnic difference in skin wrinkling manifestation between German and Japanese women could be explained by differences in sun exposure, among other factors.

A reproducible scale to assess skin aging in a population where sun exposure is high and skin phototype is dark is of great importance for clinical practice and for research.

Another study by Vierkötter et al. [8] demonstrated that the occurrence of pigments spots and wrinkles is different between Caucasians and East Asians.

The SCINEXA score was low compared to that obtained in Longo et al. who demonstrated that for the elderly, this mean score was 28.7 with SD of 6.0 [9].

In accordance with the existing literature we assume that most of the people we studied have been exposed to sun almost their lives and this will for sure cause extrinsic skin aging.

The intraclass correlation coefficient (ICC) accounts for both the consistency of performances from test to retest (within-subject change) as well as the change in average performance of participants as a group over time [10, 11]. The intraclass correlation coefficient (ICC) is the mathematical equivalent of the weighted kappa for ordinal data [12]. Hence, there is evidence for the repeatability of construct measurements between time points.

Most participants were women, which could be a limitation of the study. Another limitation of this study was its small sample size. Although the findings were good, perhaps the use of a larger sample size could have resulted in narrower confidence intervals and therefore better reproducibility estimates. The study could be further tested using a more diverse ethnic mix, in both urban and rural settings, as well as in other Ecuadoreans. However, this study is probably the first report to establish the reproducibility of the SCINEXA score in the country.

## Conclusions

This study provides evidence for test-retest reproducibility, and response stability of the SCINEXA scale in non-Caucasian people living in a specific geographical area from South America, but not for the entire country. Formal training in applying this scale is always required and it should be applied by well trained researchers. It is concluded that the intra-rater and inter-observer reproducibility test was established.

The SCINEXA scores show moderate to substantial reproducibility in healthy Ecuadorean adults of Ruta Escondida de la Mitad del Mundo (“hidden route of half of the world”), where the study took place. Genetic determinants of skin phenotypes were not assessed. Future investigations are needed in this field.

## Abbreviations

CI: Confidence intraval
ICC: Intraclass correlation coefficient
IUVR: Index of ultraviolet radiation
SCINEXA: SCore for INtrinsic and EXtrinsic skin Aging
UDLA: Universidad de Las Américas
UV: Ultraviolet
